# Genetically determined telomere length and its association with chronic obstructive pulmonary disease and interstitial lung disease in biobank Japan: A Mendelian randomization study

**DOI:** 10.1016/j.heliyon.2023.e23415

**Published:** 2023-12-06

**Authors:** Yanan Zhu, Yaxian Meng, Yasi Zhang, Ida K. Karlsson, Sara Hägg, Yiqiang Zhan

**Affiliations:** a Department of Epidemiology, School of Public Health (Shenzhen), Sun Yat-Sen University, Shenzhen, China; b Department of Medical Epidemiology and Biostatistics, Karolinska Institutet, Stockholm, Sweden; c Institute of Environmental Medicine, Karolinska Institutet, Stockholm, Sweden

**Keywords:** Chronic obstructive pulmonary disease, Interstitial lung disease, Telomeres length, Mendelian randomization, Asian population

## Abstract

**Importance:**

Chronic obstructive pulmonary disease (COPD) and interstitial lung disease (ILD) have been linked to shorter telomere length (TL). While understanding this association has critical clinical implications for respiratory diseases, previous studies exploring these associations were conducted in European populations. The present study aims to investigate this relationship in an Asian population.

**Objective:**

To examine the causal relationship between leukocyte TL and COPD and ILD in an Asian population.

**Design:**

Setting, and Participants: We used a genome-wide association study summary statistics-based two-sample Mendelian randomization (MR) design to investigate the association between leukocyte TL, genetically predicted by nine single-nucleotide polymorphisms and the risk of COPD and ILD. Participants were Japanese individuals enrolled in the Biobank Japan Project, including 3315 COPD patients and 806 ILD patients.

**Exposure:**

Leukocyte TL was genetically predicted by nine single-nucleotide polymorphisms.

**Results:**

The inverse-variance weighted estimates showed a significant inverse association between leukocyte TL and COPD (odds ratio [OR] = 0.78; 95 % confidence interval [CI]: 0.64, 0.95; P = 0.01) and ILD (OR = 0.29; 95 % CI: 0.14, 0.61; P = 0.001), respectively. All sensitivity analyses yielded consistent results. The MR-Egger regression intercept test showed no evidence of horizontal pleiotropy (P_intercept_: COPD, 0.56; ILD: 0.70).

**Conclusion:**

and Relevance: Our findings suggest that leukocyte telomere shortening may causally increase the risk of COPD and ILD. These results highlight the potential importance of TL for these respiratory diseases.

## Abbreviations

COPD: chronic obstructive pulmonary disease
ILD: interstitial lung disease
AT2: alveolar type II epithelial
GWAS: genome-wide association study
TL: telomere length
SNP: single-nucleotide polymorphism
SCHS: Singapore Chinese Health Study
IVW: inverse-variance weighting
MR: Mendelian randomization
IPF: idiopathic pulmonary fibrosis

## 1 Introduction

Telomeres, characterized by repetitive DNA sequences (TTAGGG)n, are located at the terminal ends of chromosomes. Their primary function is to safeguard chromosomes and significantly contribute to cellular longevity [1,2]. The inherent mechanism of polymerase, which inhibits DNA end elongation, results in a marginal reduction of telomeric DNA with each cellular division. Consequently, telomere length (TL) emerges as a pivotal biomarker for cellular and organismal aging [3,4].

Chronic obstructive pulmonary disease (COPD) stands as a major public health issue and ranks third in global causes of mortality [5,6]. Accumulated clinical and epidemiological evidence postulates that the etiology of COPD is potentially rooted in chronic inflammation and oxidative stress. These elements might instigate accelerated aging, leading to premature cellular senescence [[7], [8], [9]]. Corroborating this notion, several studies have established a correlation between TL and COPD [[10], [11], [12], [13], [14], [15]], However, the definitive role of TL, in the context of an aging biomarker, in COPD pathogenesis remains ambiguous.

Further complicating the landscape of age-associated disorders is interstitial lung disease (ILD), typified by inflammation and fibrosis in the lung parenchyma [[16], [17], [18], [19]]. The direct influence of aging on lung fibrosis initiation remains elusive [20]. Nevertheless, prevailing evidence suggests that telomere attrition-induced DNA damage responses might compromise the regenerative capacity of alveolar type II epithelial cells (AT2), potentially triggering apoptosis, senescence, or a combined phenotype [21,22]. Hence, postulating a pathogenic role for TL in ILD is plausible.

**Fig. 1:**
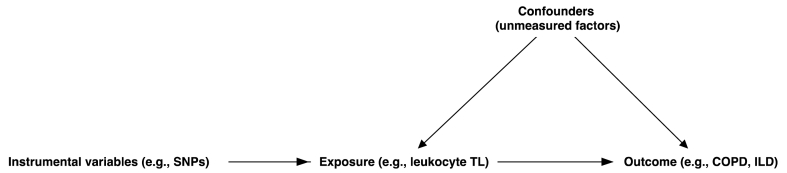
**Directed acyclic graph for the design and assumptions of Mendelian randomization method.** Mendelian randomization analyses use instrumental variables to infer causal relationships between exposures and outcomes. SNPs: single nucleotide polymorphisms; TL: telomere length; COPD: chronic obstructive pulmonary disease; ILD: interstitial lung disease.

It is imperative to highlight the predominant focus of extant studies on European cohorts, resulting in a knowledge gap concerning these diseases in Asian demographics. The present study endeavors to bridge this gap, concentrating on an Asian population, thereby augmenting the global comprehension of COPD and ILD. To delve deeper into the putative causal nexus between TL and COPD or ILD, we advocate the employment of Mendelian randomization (MR). MR, an instrumental variable methodology, is tailored to ascertain causality between phenotypes, exemplified by leukocyte TL as exposure and COPD or ILD as potential outcomes [23]. Underpinning MR is Mendel's law of independent assortment, emphasizing the independent inheritance of traits. Consequently, MR's random allele segregation delineates distinct exposure and control cohorts, ensuring a balanced distribution of unobserved confounders [24]. Given the immutable nature of an individual's genetic constitution post-conception, MR remains largely impervious to confounding or reverse causation biases [25]. Three cardinal conditions are essential for a successful MR study: 1) a demonstrable association between the instrumental variables and the exposure; 2) the absence of an independent effect of these variables on the outcome, barring their influence on the exposure; and 3) the non-existence of a shared causative pathway between the instrumental variables and the outcome (Fig. 1).

**Fig. 2:**
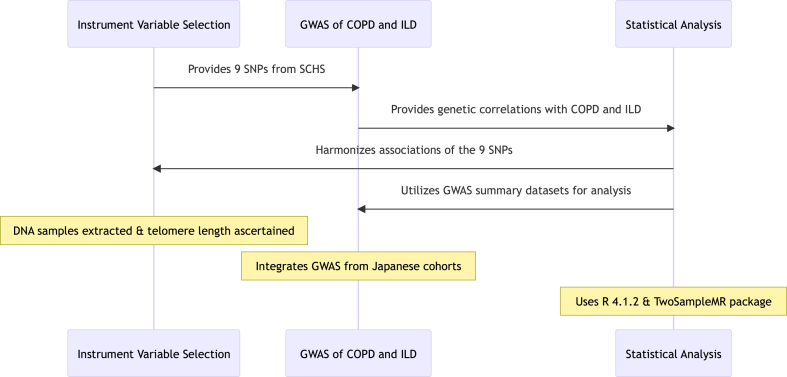
**Methodology Diagram.** Instrument Variable Selection: This step involves the selection of nine Single Nucleotide Polymorphisms (SNPs) from the Singapore Chinese Health Study (SCHS) to be used as instrumental variables for leukocyte TL; GWAS of COPD and ILD: This step integrates a GWAS from Japanese cohorts, aligning with the nine SNPs from the Singaporean Chinese population. It provides genetic correlations with COPD and ILD; Statistical Analysis: This step involves the harmonization of associations of the nine SNPs across two GWAS summary datasets and the subsequent statistical analysis.

The study's primary objective is to examine the causal impact of leukocyte TL on COPD and ILD, employing genetic association data from two distinct GWAS centered on Asian populations, all encapsulated within the MR framework (Fig. 2).

## 2 Materials and methods

### 2.1 Instrument variable selection

Table 1 presents nine single nucleotide polymorphisms (SNPs) employed as instrumental variables for leukocyte TL. These SNPs were selected from a GWAS involving 23,096 participants from the Singapore Chinese Health Study (SCHS) [26]. The inclusion of these SNPs was predicated on their significant association (p < 5 × 10^−8^) with leukocyte TL, affirming their validity as instrumental variables for our Mendelian randomization analysis. However, these SNPs were not validated in an independent Asian population.

**Table 1:** The nine instrumental SNPs and their associations with leukocyte telomere length.

SNP	Chromosome	Position (hg37)	Effect allele	Other allele	β	se(β)
rs3219104	1	226,562,621	C	A	0.074	0.009
rs2293607	3	169,482,335	C	T	−0.120	0.009
rs10857352	4	164,101,482	G	A	0.058	0.010
rs7705526	5	1,285,974	A	C	0.118	0.009
rs7776744	7	124,599,749	G	A	−0.058	0.009
rs12415148	10	105,680,586	C	T	0.204	0.020
rs227080	11	108,247,888	G	A	−0.060	0.009
rs41293836	14	24721327	T	C	0.233	0.017
rs41309367	20	62309554	T	C	−0.058	0.010

SNP: single nucleotide polymorphism.

For genotyping, DNA samples were extracted from participants' peripheral blood using the DNA Blood Kit (Qiagen, Valencia, CA). Subsequently, relative telomere length, defined as the telomere (T) to albumin (S) gene copy ratio relative to a reference from 77 SCHS participants, was ascertained via monochrome multiplex quantitative PCR.

The relative telomere length was defined as the ratio of the number of telomere (T) gene copies to the number of albumin (S) gene copies, relative to a reference sample. This reference sample was obtained from 77 participants in the SCHS. The measurement of this ratio was conducted using monochrome multiplex quantitative PCR, a technique that allows for the simultaneous amplification and quantification of multiple DNA sequences in a single reaction. This method provides a reliable and efficient way to measure relative telomere length, thereby facilitating our understanding of its association with the selected SNPs.

### 2.2 GWAS of COPD and ILD

Despite the proliferation of genetic research, a preponderance of samples is derived from European individuals, engendering a paucity of global ethnic and genetic diversity [[27], [28], [29], [30], [31]]. This could constrain our comprehension of disease mechanisms in non-European populations. Moreover, this disproportionate representation could potentially lead to biased predictions. To illustrate, polygenic risk scores, drawn from large-scale genetic studies involving European populations [[27], [28], [29], [30], [31], [32], [33], [34], [35], [36], [37]], demonstrate a robust predictive power for clinical outcomes within the same ethnic group but exhibit a significantly reduced predictive accuracy when applied to non-European populations.

In an effort to mitigate this bias, our study incorporates a recent GWAS conducted on Japanese populations, intending to correspond with nine preselected SNPs from Singaporean Chinese populations. The GWAS sourced genetic associations with COPD and ILD from a vast pool of 179,660 patients engaged in the Biobank Japan Project (BBJ) in conjunction with 32,793 population-based controls [38]. The Biobank Japan Project is a registry of patients diagnosed with any of 47 target common diseases and was launched in 2003. Project participants were recruited between June 2003 and March 2008 from 66 hospitals, which consisted of 12 cooperating medical institutions located throughout Japan. The patients who were not of East Asian descent were excluded. After the baseline survey, information was collected from participants once a year until March 2013.

The clinical definition of COPD employed in this study entails patients displaying a 1-s rate, also known as the ratio of the first second of forced expiration to the forced vital capacity, less than 70 %. Moreover, patients included under this definition have all alternative causes of airflow obstruction systematically ruled out. These individuals have also received a formal diagnosis of COPD based on the Global Initiative for Chronic Obstructive Lung Disease (GOLD) standards and are undergoing medical treatment.

The classification of ILD includes patients whose diagnosis has been substantiated either by pathological findings or high-resolution computed tomography (CT) of the chest. Alternatively, patients may have previously received a diagnosis of interstitial pneumonia or pulmonary fibrosis and are currently being treated with medication.

The data (Table 2), available publicly and without any access restrictions, can be found on JENGER (http://jenger.riken.jp/en/) and the National Bioscience Database Center (NBDC; https://humandbs.biosciencedbc.jp/en/) Human Database (Research ID: hum0014).

**Table 2:** Sample sizes for the GWAS on chronic obstructive pulmonary disease (COPD) and interstitial lung disease (ILD), respectively.

Disease name	Cases	Controls	Total
COPD	3315	201,592	204,907
ILD	806	211,647	212,453

### 2.3 Statistical analysis

Prior to analysis, we harmonized associations of the nine SNPs across two GWAS summary datasets [39]. In the principal phase of our investigation, we utilized a fixed-effect inverse-variance weighted (IVW) method to estimate causal effects. This particular method, owing to its inherent statistical power, holds an advantage over other estimators when all instrumental SNPs are valid [39].

To complement the primary analysis, we performed several sensitivity analyses as well. We implemented a weighted median approach to estimate the mean pleiotropic effect of genetic variants. Additionally, we utilized the MR-Egger regression and the weighted mode method, the latter of which was specifically employed when up to half of the genetic variants were rendered invalid [[40], [41], [42]]. In an attempt to account for any inherent heterogeneity in the data, we employed a multiplicative random-effect model of the IVW method for comparative analysis of results. Furthermore, we embarked on a directional pleiotropy test with the aim of examining the potential causation of TL with COPD and ILD.

All computations were executed in R 4.1.2 and the *TwoSampleMR* package [43]. Our analyses were based solely on publicly available summary statistics of GWAS, abstaining from the use of any individual-level data. An ethical permit is therefore not required.

## Results

In this study, we used the GWAS data of leukocyte TL, COPD, and ILD for a two-sample MR analysis, based on the Singapore Chinese and Japanese. It is acknowledged that all of them are the East Asian descent and have more similar genomes compared with those of other ethnic. In addition, the participants recruited to the BBJ project are non-overlapped to the participants that generated the GWAS data of leukocyte TL.

### 3.1 Causal association of leukocyte TL with COPD

**Fig. 3:**
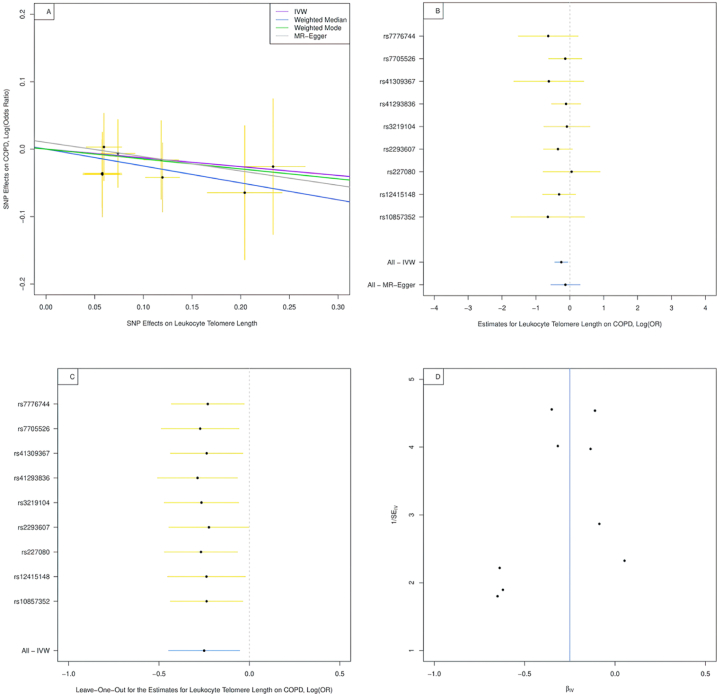
Combination plot on COPD analysis. **Panel A. Scatter plot for the genetic association of selected instrumental SNPs on leukocyte telomere length and COPD.** IVW: inverse-variance weighted; MR: Mendelian randomization; COPD: chronic obstructive pulmonary disease. The horizontal axis represents the effects of each genetic variant on leukocyte telomere length and the vertical axis denotes the effects of each genetic variant on COPD. The orange lines around the solid black points are the corresponding confidence intervals for the effects. The slopes of solid lines represent the estimates from IVW, weighted median, weighted mode, and MR-Egger regression analyses. **Panel B. Single SNP plot for the estimates for Leukocyte Telomere Length on COPD.** IVW: inverse-variance weighted, MR: Mendelian randomization, COPD: chronic obstructive pulmonary disease. **Panel C. Leave-one-out analysis for the estimates for leukocyte telomere length on COPD.** IVW, inverse-variance weighted; COPD, chronic obstructive pulmonary disease. **Panel D. Funnel Plot for the SNPs on COPD.** IV: instrumental variable, COPD: chronic obstructive pulmonary disease.

In our analysis focused on the causal effect of leukocyte TL on COPD, all nine instrumental SNPs were identifiable within the GWAS of COPD. Consequently, these SNPs were subjected to analysis within our MR models. The genetic associations of these SNPs with leukocyte TL and COPD have been visually represented in Fig. 3A as a scatter plot.

Through the application of the IVW method, our study revealed a statistically significant inverse association between leukocyte TL and COPD, with an odds ratio (OR) of 0.78 (95 % confidence interval (CI): 0.64,0.95, *P* = 0.01). However, it is noteworthy that these findings were largely consistent with the supplementary analyses, albeit with wider confidence intervals, as illustrated in Table 3.

**Table 3:** Association of genetically predicted leukocyte telomere length with chronic obstructive pulmonary disease.

Method	OR	95 % CI	*P*-value
Inverse-variance weighted	0.78	0.64, 0.95	0.01
MR-Egger regression	0.88	0.57, 1.35	0.57
Weighted median	0.81	0.64, 1.03	0.08
Weighted mode	0.82	0.60, 1.12	0.25

OR: odds ratio; CI: confidence interval.

We employed the MR-Egger test to explore the presence of directional pleiotropy in our data. The results suggested a lack of evidence supporting directional pleiotropy (with an intercept of −0.013 and a *P*-value of 0.56). Furthermore, our analysis also did not reveal any evidence pointing toward the existence of heterogeneity within our data.

As per our single SNP Plot depicted in Fig. 3B,C, and 3D, the association between the nine SNPs analyzed individually and the outcome was not statistically significant. Interestingly though, when combined, these SNPs manifested a statistically significant result via the IVW method. Nonetheless, this finding was not echoed by the MR-Egger regression approach, thereby introducing an element of variability into our results.

### 3.2 Causal association of leukocyte TL with ILD

**Fig. 4:**
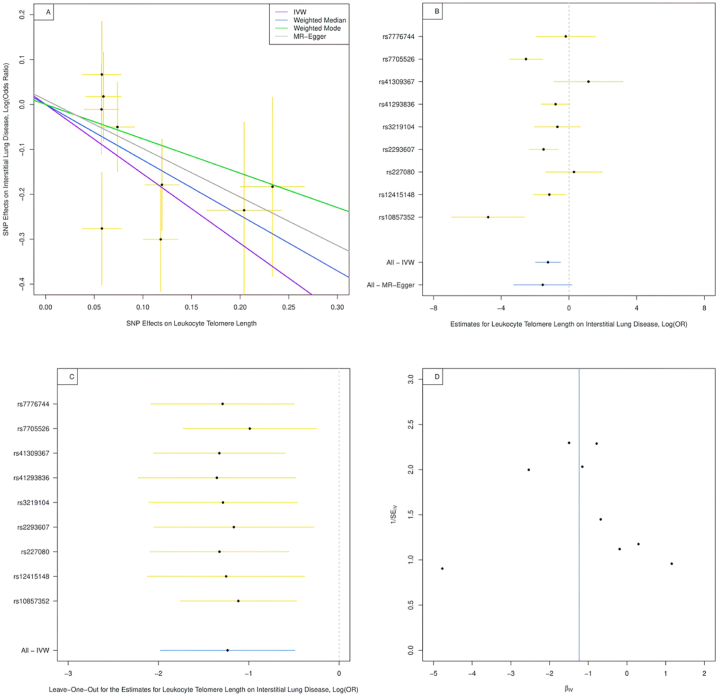
Combination plot on ILD analysis. **Panel A. Scatter Plot for the Effects of SNPs on Leukocyte Telomere Length and ILD.** IVW: inverse-variance weighted, MR: Mendelian randomization, ILD: interstitial lung disease. The horizontal axis represents the effects of each genetic variant on leukocyte telomere length and the vertical axis denotes the effects of each genetic variant on ILD. The orange lines around the solid black points are the corresponding confidence intervals for the effects. The slopes of solid lines represent the estimates from IVW, weighted median, weighted mode, and MR-Egger regression analyses. **Panel B. Single SNP Plot for the estimates for Leukocyte Telomere Length on ILD.** IVW: inverse-variance weighted, MR: Mendelian randomization, ILD: interstitial lung disease. **Panel C. Leave-One-Out Analysis for the estimates for Leukocyte Telomere Length on ILD.** IVW: inverse-variance weighted, ILD: interstitial lung disease. **Panel D. Funnel Plot for the SNPs on ILD.** IV: instrumental variable, ILD: interstitial lung disease.

A scatter plot was designed to delineate the effects of various SNPs on leukocyte TL and ILD, as depicted in Fig. 4A. Notably, the IVW method, the weighted median, and the weighted mode approach were implemented to substantiate the statistical significance of these observations (as detailed in Table 4). A consistent inference drawn from these methods was the inverse association between leukocyte TL and the risk of ILD - longer leukocyte TL correlated with a reduced risk of developing ILD. However, this correlation didn't hold its statistical significance when explored using the MR-Egger regression approach (OR = 0.21, 95 % CI:0.04,1.20, *P*-value = 0.12).

**Table 4:** Association of genetically predicted leukocyte telomere length with interstitial lung disease.

Method	OR	95 % CI	*P*-value
Inverse-variance weighted	0.29	0.14, 0.61	0.001
MR-Egger regression	0.21	0.04, 1.20	0.12
Weighted median	0.34	0.19, 0.60	0.0002
Weighted mode	0.36	0.18, 0.70	0.02

OR: odds ratio; CI: confidence interval.

On the individual level, four SNPs (rs7705526, rs2293607, rs12415148, rs10857352) were found to be statistically significant as shown in the single SNP plot (Fig. 4B and C). When these SNPs were pooled with five others and analyzed using the IVW method, statistical significance was achieved. In contrast, the remaining five SNPs, as well as the MR-Egger regression approach, did not reach a level of statistical significance, indicating that these SNPs may not substantially contribute to leukocyte TL or ILD risk.

Further validation via a leave-one-out analysis affirmed these observations, implying that the results in ILD were not dominated by any single SNP. These findings were graphically represented in a funnel plot (Fig. 4D). Further, we applied a multiplicative random-effects model for the IVW method and found a significant β value of −1.23 (*P*-value = 0.001). The latter illustrates a negative correlation, reaffirming the inverse relationship between leukocyte TL and ILD. Finally, no evidence of directional pleiotropy was observed in the MR-Egger regression analysis (intercept: 0.035, *P*-value = 0.70). This absence of pleiotropy signifies that the SNPs are likely affecting ILD predominantly through their influence on leukocyte TL, not via other biological pathways.

We additionally performed reverse MR analysis to examine if COPD and ILD were potentially causing telomere shortening. The analyses did not support significant associations of genetically predicted COPD and ILD with TL for the IVW methods (***β*** = 0.002, P = 0.78 and ***β*** = −0.004, P = 0.69).

## 4 Discussion

In this study, we applied an MR method to examine whether there was a causal association between leukocyte TL and the risk of COPD and ILD. Nine SNPs that were identified from SCHS were used as instrumental variables that were independent and available in BBJ. All results of IVW, MR-Egger regression, weighted median, and mode-based method provided evidence that genetically determined leukocyte TL had a causal effect on COPD (OR:0.78, 95 % CI: 0.64, 0.95, p = 0.01) and ILD (OR: 0.29, 95 % CI:0.14, 0.61, p = 0.001). To be specific, the longer the leukocyte TL, the lower the risks of developing COPD and ILD. Our result of the primary analysis of IVW is robust, unbiased, and reliable on account of neither substantial heterogeneity nor evident pleiotropy.

To our knowledge, this study is the first to provide evidence supporting the potential causal effect of longer leukocyte TL on two pulmonary diseases, COPD and ILD, in an Asian population. This study adds to previous observational studies that provide evidence of shorter LTL in patients with COPD. Some case-control studies [1,2] have found both young and old patients with severe COPD may have shorter TL. A three-year cohort study also found that patients with COPD have accelerated telomere shortening [3], which is consistent with this current study. A variety of environmental and behavioral factors (tobacco smoking, diet, stress, air pollution) might have an influence on leukocyte telomeres [4]. Many of these factors have been shown to be associated with a higher risk of COPD, which provides a more theoretical basis for our research. Contrary to our findings, Duckworth et al. found no association between leukocyte TL and COPD using European ancestry data from the UK Biobank in their MR method. This discrepancy could be attributed to the differential genetic architecture and the transferability of genetic findings across different populations. For instance, a study assessing leukocyte TL in a multi-ethnic cohort found that on average blacks and Hispanics had longer telomeres than whites, and these groups showed more significant differences in TL associated with age. This suggests that racial and ethnic differences in leukocyte TL could potentially explain the divergent results in these studies.

Mechanisms underlying the association of LTL with increased risk of COPD are largely unknown. We speculate that they were related to increased oxidative stress. Elevated oxidative stress is known for its pivotal role in affecting LTL and accelerating the progression of COPD. *GSTM1* is one of the genes encoding GST isoenzymes, and then protecting DNA from oxidative damage. Tanya et al. found that *GSTM1* genotypes might play a role in leukocyte telomere shortening, and thus be involved in the pathogenesis of COPD.

Our study, using the multiplicative random-effects model for the IVW method, suggested a causal association between leukocyte TL and ILD. This is consistent with previous observational studies [7], as well as a previous MR analysis that inferred shorter telomeres cause IPF, the most common subgroup of ILD. The incidence of IPF and other fibrotic ILDs increases with age, suggesting a possible aberration of the normal aging process of the lung [[16], [17], [18], [19]]. Furthermore, shorter TLs were observed in AT2 cells of IPF lungs compared to controls, and AT2 cell TL was longer in non-fibrotic areas than in fibrotic areas of the same biopsy. While the nature of telomere abnormalities and their role in the biology of lung fibroblasts remain largely unknown, our study, in conjunction with previous research, strongly suggests a causal relationship between TL and ILD. Therefore, this study adds to the growing evidence demonstrating that TL is a prognostic biomarker of ILD. The pathogenesis of ILD may be related to inherited mutations in telomerase and other telomere maintenance genes. Studies have shown that 90 % of individuals who carry these inherited mutations develop chronic lung disease [8]. More studies have found that TL is associated with the progression and survival of ILD [9,10], and individuals with short telomeres and/or known telomere-related mutations have more rapid disease progression and shorter lung transplant-free survival [11,12].

Our study has several strengths. Firstly, we utilized publicly available GWAS data for leukocyte TL, COPD, and ILD, which included large sample sizes, thereby enhancing the precision and statistical power of our evaluation. Secondly, the MR method used in our study is less biased than general observational studies, providing a more accurate estimation of causal associations. Furthermore, we leveraged the two-sample MR method, which utilized GWAS summary levels for leukocyte TL, COPD, and ILD derived from two independent populations. However, our study also has limitations. We did not directly evaluate leukocyte TL. We assumed that the nine selected SNPs were strongly associated with leukocyte TL. Additionally, our analyses were based on a linear assumption, and we did not account for potential non-linearity. We did, however, apply MR-Egger regression to evaluate potential bias caused by genetic pleiotropy. Our results are based on genetically predicted TL measured only in leukocytes, not other cell types such as alveolar cells. Despite this, leukocyte TL can be considered a good proxy for overall TL, given previous reports of high correlation across TL measured in different tissue types. Further, emerging technologies such as artificial intelligence and telecommunications could be applied to recruit study participants and to test SNPs in a more efficient way. Additionally, leukocyte TL or genetically predicted TL could be used as a screening tool for COPD and ILD detection. Lastly, our results are based on GWAS data from individuals of Japanese ancestry, limiting the generalizability of our findings to other ethnic populations. Future works could focus on COPD and ILD prediction modeling with TL using individual-level data with long follow-ups in a diverse population.

In conclusion, our study demonstrated a causal association between leukocyte TL and both COPD and ILD in an Asian population. We found that shorter leukocyte TL is associated with a higher risk of developing COPD and ILD. Further research is needed to replicate our findings and to elucidate the molecular mechanisms underlying these relationships.

## Ethics statement

This study strictly adheres to ethical guidelines and legal regulations to ensure the rights and privacy of all participants are fully protected. As the research is solely based on publicly available GWAS summary statistics, it does not involve any individual-level data or any research activities related to human or animal samples. Therefore, the study did not require approval from an ethics review board.

## Data availability statement

All data utilized in this study are publicly available. Specifically, the GWAS summary statistics used for the research can be found on JENGER (http://jenger.riken.jp/en/) and the National Bioscience Database Center (NBDC; https://humandbs.biosciencedbc.jp/en/) Human Database (Research ID: hum0014). These data are publicly available and free to use without any access restrictions.

## CRediT authorship contribution statement

**Yanan Zhu:** Writing – original draft, Formal analysis. **Yaxian Meng:** Writing – review & editing, Writing – original draft. **Yasi Zhang:** Writing – original draft, Validation, Formal analysis. **Ida K. Karlsson:** Writing – review & editing. **Sara Hägg:** Writing – review & editing, Conceptualization. **Yiqiang Zhan:** Writing – review & editing, Supervision, Software, Resources, Project administration, Methodology, Data curation, Conceptualization.

## Declaration of competing interest

The authors declare that they have no known competing financial interests or personal relationships that could have appeared to influence the work reported in this paper.
